# 105 years of phoniatry

**DOI:** 10.1590/S1808-86942010000300001

**Published:** 2015-10-20

**Authors:** Sandra Irene Cubas de Almeida

**Affiliations:** PhD in Medicine – UNIFESP Coordinator of the Phoniatry Department-ABORLCCF

In the history of medicine it is hard to pinpoint the exact time when medical specialty sprouts. Phoniatry, however, is one of these fields that have a specific landmark to its birth. 105 years ago, in the University of Charité in Berlin, the course of phoniatry was created, stemming from the thesis of a Professor from the Department of Internal Medicine: “Die Atembewegungen in ihrer Beziehung zu den Sprachstörungen” (Respiratory Movements and Their Association with speech disorders) by Hermann Gutzmann.

Nonetheless, everything has a starting point and Gutzman's Biography discussed how family and stimuli influence the actions of an individual and his/her most intimate interests.

Gutzmann was the son of Albert Gutzmann, who taught deaf people and, one of the first scholars of communication disorders to try to establish a scientific approach to rehabilitate his students. Among his publications we list “Das Stottern und seine gründliche Beseitigung durch ein methodisch geordnetes um praktisch erprobtes Verfahren”, which discusses dysfluency. Hermann studies medicine and writes his doctorate paper also discussing phoniatry.

After that, the scientific methodology with the basis of medical knowledge becomes a necessary and logical pathway in the diagnosis and treatment of disorders which affect communication. As we mentioned before, the course was first introduced in the department of internal medicine and later it was transferred to the Department of Otorhinolaryngology. Gutzmann always advocated interdisciplinarity, considering all the fields of knowledge which are important for understanding and approaching disorders of the voice, speech, language and hearing. His school was characterized by having an anatomical and physiological basis.

Emil Fröschels, had ties with the Vienna School, he is one of the founders of the IALP (International Association of Speech and Hearing Therapists and Phoniatrists) adding a psychological approach to Phoniatry.

In Brazil we have two personalities who introduced phoniatry to our medical practice: Pedro Bloch and Mauro Spinelli. In Argentina, Qirós had an extremely relevant scientific production in the development of this field.

At the moment, Phoniatry is a very specific field of practice, belonging exclusively to Otolaryngologists. Besides having more knowledge about it, considering its complexity, it is desirable that the phoniatrist's training encompasses specific courses in the field of human sciences, such as linguistics, philosophy and pedagogy. Thus, when the phoniatrist chooses his/her path, he/she will have a long and continuous journey pursuing a better practice.

The phoniatry department of the ABORLCCF, created under the pioneering steering of Prof. Ricardo Bento, has held its first program and has gathered the colleagues who practice Phoniatry. Our efforts are concentrated in divulging knowledge and stimulating ENTs to be increasingly more interested in this field. We are gathering the scientific material from the first program, in which we discussed the main needs to make it all available through our journal. I hope everyone enjoys and uses it.

We would like to thank the Editor, Prof. João Mello and our Chairman, Prof. Ricardo Bento.

